# Assistance and Therapy Dogs Are Better Problem Solvers Than Both Trained and Untrained Family Dogs

**DOI:** 10.3389/fvets.2020.00164

**Published:** 2020-03-31

**Authors:** Fabricio Carballo, Camilla María Cavalli, Márta Gácsi, Ádám Miklósi, Enikő Kubinyi

**Affiliations:** ^1^Department of Ethology, Institute of Biology, ELTE Eötvös Loránd University, Budapest, Hungary; ^2^Instituto de Ciencias Biológicas y Biomédicas del Sur (INBIOSUR), Departamento de Biología Bioquímica y Farmacia, Universidad Nacional del Sur (UNS)- Consejo Nacional de Investigaciones Científicas y Técnicas (CONICET), Bahía Blanca, Argentina; ^3^Grupo de Investigación del Comportamiento en Cánidos (ICOC), Instituto de Investigaciones Médicas (IDIM), Consejo Nacional de Investigaciones Científicas y Técnicas, Universidad de Buenos Aires, Buenos Aires, Argentina; ^4^MTA-ELTE Comparative Ethology Research Group, Budapest, Hungary

**Keywords:** human-animal interaction, canine-cognition, persistence, gazing, unsolvable task, working dogs

## Abstract

When faced with unsolvable or difficult situations dogs use different behavioral strategies. If they are motivated to obtain rewards, they either try to solve the problem on their own or tend to interact with a human partner. Based on the observation that in problem situations less successful and less perseverant dogs look more at the humans' face, some authors claim that the use of social strategies is detrimental to attempting an independent solution in dogs. Training may have an effect on dogs' problem-solving performance. We compared the behavior of (1) untrained, (2) trained for recreational purposes, and (3) working dogs: assistance and therapy dogs living in families (*N* = 90). During the task, dogs had to manipulate an apparatus with food pellets hidden inside. We measured the behaviors oriented toward the apparatus and behaviors directed at the owner/experimenter, and ran a principal component analysis. All measures loaded in one factor representing the use of the social strategy over a more problem-oriented strategy. Untrained dogs obtained the highest social strategy scores, followed by dogs trained for recreational purposes, and assistance and therapy dogs had the lowest scores. We conclude that assistance and therapy dogs' specific training and working experience (i.e., to actively help people) favors their independent and more successful problem-solving performance. General training (mainly obedience and agility in this study) also increases problem-oriented behavior.

## Introduction

Problem-solving behaviors involve a diverse set of cognitive processes, such as perception, learning, memory and decision making, among others (1, 2).

Several studies have focused on dogs' problem-solving abilities using a wide variety of tasks (e.g., puzzle boxes in Frank and Frank (3) and Marshall-Pescini et al. (4); unsolvable task in Miklósi et al. (5); string pulling in Osthaus et al. (6); interactive dog toy in Shimabukuro et al. (7). Different kinds of tasks require different skills, thus allowing the thorough study of the diverse strategies that dogs use to solve problems [see e.g., Polgár et al. (8)]. While some studies focus on the manipulation of the physical environment, others analyse social strategies, including communicative interactions. With regard to the latter, dogs' gazing behavior has received the most attention. One frequently used protocol to assess dogs' communicative intents toward people is the so-called unsolvable task. In this situation, dogs try to obtain a reward from an apparatus that cannot be opened. When faced with this problem, most dogs tend to gaze at their owners, which can be interpreted as a referential request for assistance by the human partner [e.g., Miklósi et al. (5), for a review see Cavalli et al. (9)].

Dogs' selection for socio-cognitive abilities during the domestication process might have had a detrimental effect on their physical cognition (3). This hypothesis has been supported by several comparative studies in which dogs privileged the use of social strategies such as gazing to the human face, while wolves spent more time manipulating an apparatus and were thus more successful in solving the problem (10, 11). However, other authors have highlighted that this discrepancy in the performance of the two species may not be (only) due to differences in their ability to solve physical problems, but other factors, such as motivation and persistence (12–14), and vague definitions (15). Persistence is a reliable predictor of problem-solving ability, and might be linked to trial and error learning strategies (16). In this regard, persistence has been operationally defined as the time spent interacting with an apparatus (17). Accordingly, those individuals that persist longer in their problem-solving attempts are more likely to solve a problem than those that give up earlier [e.g., (16)].

Several other factors appear to influence dogs' problem-solving abilities, including their relationship with humans (18), their living conditions (19, 20), and their breed. For example, compared to Siberian huskies, border collies looked more at the owner in an unsolvable problem situation (21), and herding dogs tended to look more at the person than working and mastiff like dog breeds when confronted with a puzzle box (22). However, herding dogs did not interact more with the apparatus than other breed groups in this study, and when taking into consideration both breed and training experience, training had a major influence on dogs' orientation to the apparatus (22).

In line with this, many studies have focused on the role of training experience. This is of particular relevance, considering the importance of training in working dogs' performance and the increased number of tasks in which dogs participate nowadays. For instance, Marshall-Pescini et al. (4) tested the performance of untrained family dogs and highly trained family dogs that participated in different activities (i.e., agility, schutzhund, retrieving, search and rescue, freestyle performances). All dogs were exposed to a commercial feeding box which could be opened by pressing a paw pad or nosing the lid. While untrained dogs spent significantly more time looking at either the experimenter or their owner; trained dogs interacted significantly longer with the apparatus and were more successful in opening it. Marshall-Pescini et al. (22) observed similar results using the same apparatus, as dogs with training experience (i.e., agility, police, search and rescue, and man-trailing) were more successful in the task and looked less to people than untrained dogs. It is important to note that in both of the aforementioned studies trained dogs' groups were heterogeneous given that the subjects differed in the types of training they received and their everyday experiences. While some dogs were trained working dogs, others were trained for recreational or sporting purposes such as agility. Thus, to disentangle the relative effects of training for recreational purposes and for specific work, we aimed to compare the performance of dogs trained for assistance and therapy work with family dogs which had been trained for recreational purposes (see subjects' details). Assistance and therapy dogs differed from trained family dogs in the purpose of their training, their everyday tasks and in the methods of training.

Range et al. (23) carried out a similar experiment, using a wooden box with a handle which could be opened by pushing it down with the mouth or a paw. In line with previous results, trained dogs (i.e., agility and search and rescue) spent more time interacting with the apparatus and were able to open it significantly more often than untrained ones (23). On the contrary, Brubaker and Udell (24) found no significant differences between search and rescue dogs and untrained family dogs in gazing or persistence in a similar task. However, significantly more search and rescue dogs opened the container when they received encouragement (24). The divergence between these studies may be related to differences in the training the dogs from each sample had received [i.e., agility and rescue dogs in Range et al. (23); only rescue dogs in Brubaker and Udell (24)]. Furthermore, the encouragement in Brubaker and Udell (24) may have also influenced the results and this difference in the protocols hinders a straightforward comparison. All in all, results regarding the effects of training on dogs' problem-solving skills and strategies are contradicting. This could be due to differences in the protocols and tasks used, samples, the dogs' breed, and the training received as discussed above.

Professional working dogs represent a special group of dogs which, unlike family dogs, are specifically trained to regularly perform a specific activity such as detection of substances, search and rescue or helping disabled people, among others (25). Importantly, working dogs face a variety of cognitive challenges during their training and working activities which may influence their behavior and performance during cognitive tests. Even more, as different working roles require different sets of skills, it would be expected that working dogs vary in their performance during such tasks according to the specific activities they carry out (26). In line with this, it must be taken into account that there are variations in the goals of training, the methods employed for it and the frequency in which those abilities need to be performed, which add to the expected variability among working dogs as a whole. Thus, it is important to assess dogs with different training and working experiences to further understand how these aspects influence dogs' problem-solving skills.

In this study we focused on two types of working dogs: assistance and therapy dogs. Assistance dogs are individually trained to perform tasks for the benefit of their owner with a disability affecting everyday life situations (27). Therapy dogs participate with their owners in planned, goal-oriented therapeutic interventions directed by providers of health and human service (28). Both types of working dogs need to be sensitive to their owners' wishes, but at the same time they have to be independent in order to solve problems on their own and flexibly adjust to new scenarios.

Gácsi et al. (29) studied the interactions between assistance dogs and their owners during a carrying task. They observed joint attention during different parts of the task as well as the use of both verbal and non-verbal communication to guide the dogs' actions. In the case of a task that was impossible to perform, they observed that assistance dogs did not give up easily and were very persistent before they showed communicative signals directed at the owner (29). The results suggest that assistance dogs are not only persistent, but also able to switch between different strategies, such as communicating with the owner, if they failed in independent problem-solving.

Thus, in this study we aimed to compare the problem-solving performance of dogs with different levels of training and working experience. To this end, we tested three groups of dogs in a problem-solving task; untrained family dogs, family dogs trained for specific tasks (e.g., obedience, agility, herding), and working assistance and therapy dogs. For the sake of simplicity, we will refer to dogs working in assistance and therapy as “working dogs.” We expected working dogs to perform better at independent problem-solving and thus to obtain more food rewards than family pet dogs. Also, we expected untrained family dogs to depend more on their owners and prefer the use of a social strategy such as gazing toward people. In the case of trained family dogs, training experience may increase their independent problem-solving abilities [e.g., (4)]. If this is the case, they should behave similarly to the working dog group. Alternatively, the trainings these dogs had (mainly obedience and agility) may have not prepared them for independent problem-solving, thus their performance may be indistinguishable from that of untrained family dogs.

## Materials and Methods

### Ethical Statement

The procedures comply with national and EU legislation and institutional guidelines and in accordance with the recommendations in the International Society for Applied Ethology guidelines (www.applied-ethology.org). In Hungary, the behavioral observations conducted in this study were not identified as animal experiments by the Hungarian Animal Protection Act (“1998. évi XXVIII. Törvény,” 3. §9.), which identifies animal experiments, as this study was non-invasive. The application number of the ethical commission by the Pest County Government Office is PE/EA/2019-5/2017. Each owner filled in a consent form stating that they have been informed of the tests. Our Consent Form was based on the Ethical Codex of Hungarian Psychologists (2004).

### Subjects

We tested a total of 90 dogs between 1 and 12 years of age, of different breeds and mixed-breeds (see below). Owners volunteered to participate in the test and were recruited through the Family Dog Project database of Eötvös Loránd University, Budapest, Hungary. All dogs had been living with their owners for at least 6 months before the test. Dogs were assigned to three groups according to their work and training experience. Size, sex, and breed were balanced across groups:

1. *Untrained family dogs* had no certification exams. *N* = 30, 14 males, 16 females, mean age = 4.05, SD ± 2.74, breeds: 1 beagle, 7 border collies, 3 German shepherd dogs, 4 golden retrievers, 3 Labrador retrievers, 1 Maltese, 10 mixed, 1 English cocker spaniel.
2. *Trained family dogs* are dogs trained for recreational purposes. They had 1–4 certification exams (27 obedience, 23 agility, 11 herding, 5 guarding, 9 other: rescue dog, frisbee, dog dancing, K99). *N* = 30, 15 males, 15 females, mean age = 4.66, SD ± 2.67, Breeds: 8 Border Collies, 1 Bouvier, 1 Dobermann, 2 Golden Retrievers, 1 groenendael, 1 kelpie, 1 Labrador retriever, 2 malinois, 8 mixed, 1 mudi, 1 sheltie, 2 Hungarian vizslas, 1 Yorkshire terrier.
3. *Working dogs* worked as certified assistance or therapy dogs. assistance dogs were trained to aid individuals with disabilities by the dogs for human charity (http://kea-net.hu/). Therapy dogs were all certified trained dogs, and lived with their owners at their homes. *N* = 30, 15 males, 15 females, mean age = 4.47, SD ± 3.32, 1 Airdale terrier, 3 border collies, 1 Cavalier King Charles spaniel, 4 golden retrievers, 1 groenendael, 1 Irish setter, 2 Labrador retrievers, 1 Malinois, 8 mixed, 2 standard poodles, 1 English cocker spaniel, 4 Tervuerens, 1 Hungarian vizsla.

### Experimental Setup

**Figure 1:**
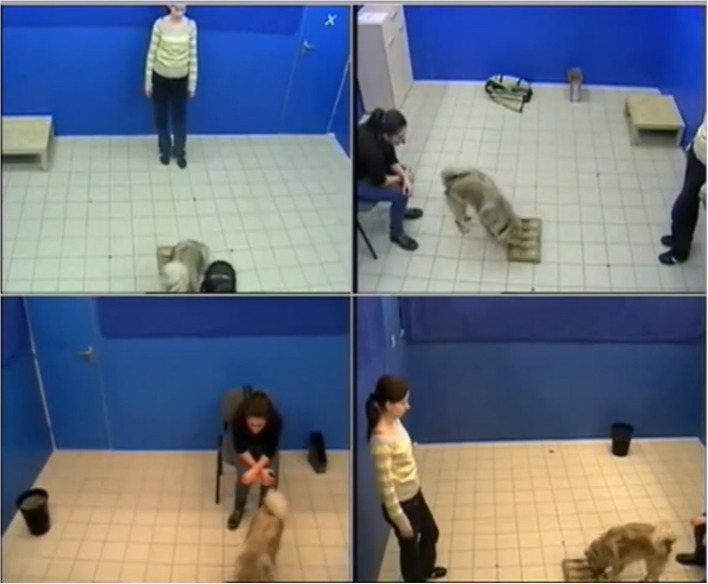
The experimental setup. Written informed consents were obtained from the individuals for the publication of this image.

All dogs had at least 1 h of fasting time before the testing. Dogs were tested in a room unfamiliar to them at the Eötvös University, Department of Ethology. Four cameras in each corner of the room videotaped all testing sessions. The room was 3 × 6 m^2^ and there was a drawer where the problem box was stored before the start of the test and a chair for the owner to sit on (Figure 1).

### Apparatus

As a problem box we used a commercial wooden dog toy (Nina Ottosson® Dog Brick) that comprised a rectangular base with eight holes where treats could be hidden. A sliding wooden brick covered eight holes on both longer sides of the toy, so dogs had to slide the covers toward the middle with their paws or nose in order to get the treats. The bricks could not be lifted. Eight pellets of dry food in the eight holes on both longer sides were used as treats. None of the dogs were familiar with the apparatus prior to the task.

### Procedure

At the beginning of the test, the owner sat on a chair holding the dog on leash. The experimenter (female, 22 years old), who was the same for all dogs, took the interactive dog toy out from the drawers, placed it on the ground, and put a pellet of dry food inside each hole. Thus, dogs were able to see the baiting. When she was ready, the experimenter stepped back, the owner released the dog and the testing began. The dog had 2 min to obtain the food pellets from the apparatus. During this period, the owner was allowed to encourage the dog to find the pellets, verbally and by pointing at the apparatus, but we forbade the use of any previously trained or known commands relevant to the task such as “catch” or “nose.” The owner could not touch the apparatus nor the dog (Figure 1). After the 2 min had elapsed, the experimenter put the toy back in the drawer. Dogs were allowed to eat only the food pellets they had recovered.

#### Behavioral Variables

We measured the duration of the vocalizations using a 0–3 score. We also measured the proportion of time dogs spent wagging their tail and the proportion of time oriented to the apparatus (including manipulating it, as gazing at the apparatus was often immediately followed by manipulation, therefore it would have been difficult to separate the two behaviors). We counted the number of times the dog gazed at the owner/experimenter, because gazing was generally a short event (just a glance) and provided more information than duration. We also counted the number of food pellets eaten after the behavior tests, on the spot. Other behavioral measures were coded from the videos using Solomon Coder (András Péter). See Table 1 for details and descriptive statistics.

**Table 1:** Descriptive statistics of the measured raw variables and factor loadings of the standardized variables.

**Behavioral variables**	**Min**	**Max**	**Mean**	**SD**	**Factor loading**
Duration of vocalization [score 1–3: (score 0: no vocalization, 1: 1–5 s, 2: 5–10 s, 3: >10 s)]					0.47
Untrained	0	3	0	1	
Trained	0	3	1	1	
Working	0	1	0	0	
Number of food pellets eaten (*n*)					−0.75
Untrained	0	4	0	1	
Trained	0	5	1	1	
Working	0	8	3	3	
Duration of orientation toward the apparatus (including manipulation, %)					−0.84
Untrained	3	90	35	25	
Trained	7	99	55	29	
Working	10	100	72	28	
Duration of tail wagging (%)					0.60
Untrained	0	98	49	35	
Trained	0	97	45	30	
Working	0	100	26	31	
Number of looking at the owner (*n*)					0.87
Untrained	3	32	16	7	
Trained	0	33	12	9	
Working	0	20	7	6	
Number of looking at the experimenter (*n*)					0.71
Untrained	0	22	6	5	
Trained	0	13	5	4	
Working	0	13	4	4	

### Statistical Analysis

We analyzed the inter-rater reliability of the variables using two-way random intraclass correlation, looking for absolute agreement between average measures. The inter-rater reliabilities were satisfactory (ICC > 0.741, *N* = 10).

After standardizing the variables, we ran principal component analysis and calculated factor scores. Cronbach alpha (CA) was used for checking the internal consistency of the factor. For investigating differences in the behavioral factor score (“social strategy” score, see below), as a function of group, sex (as fixed factors), and age (as covariate) we applied General Linear Model with Student–Newman–Keuls (SNK) *post-hoc* test, including all main effects and two way interactions. We used backward elimination to obtain the minimum adequate model. SPSS v25 (30) was used for the analyses.

## Results

Descriptive statistics of the variables and factor loadings are presented in Table 1. Standardized variables loaded on a single factor. The total explained variance of the factor was 51.5%, CA = 0.8. The factor included looking at the owner, looking at the experimenter, tail wagging, and vocalization with positive loadings while orientation toward the apparatus and number of food pellets eaten had negative loadings. We labeled this factor as “social strategy,” because high score indicated that the dog uses communicative signals toward the human partners, including gazing, vocalization, tail wagging.

**Figure 2:**
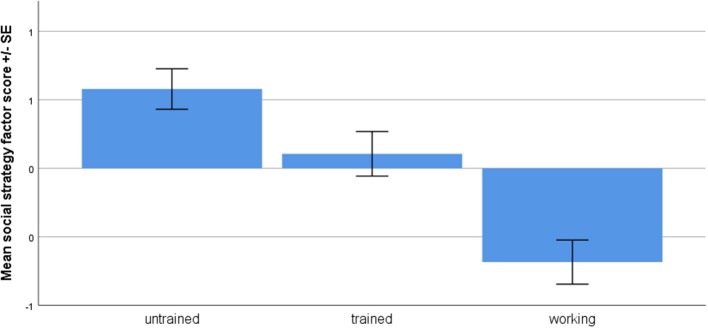
Social strategy factor scores of the three dog groups.

Only group affected the social strategy score [*F*_(2, 85)_ = 16.477, *p* < 0.001, partial eta squared = 0.275, Figure 2), age or sex had no effect and there were no interactions (all *p* > 0.05). According to the SNK *post-hoc* tests, all groups differed from each other (alpha = 0.05). Untrained dogs obtained the highest social strategy scores, trained dogs had lower scores, followed by working dogs.

## Discussion

We set out to investigate the problem-solving abilities and related behaviors of dogs with different levels of training and working experience (trained and untrained family dogs as well as working assistance and therapy dogs) in a problem-solving task. Working assistance and therapy dogs displayed a less social and more problem-oriented strategy with a higher success rate than both untrained and trained family dogs. The frequent use of social strategies (i.e., gazing) is correlated with less persistence on the task (i.e., independent manipulation of the apparatus) and consequently with lower success (17). The results are also consistent with prior literature stating that animals persisting more on their problem-solving attempts are more successful in actually solving the task (16).

As it was mentioned in the introduction, the literature is mixed regarding the effects of training on dogs' persistence and gazing behavior during problem-solving tasks. For instance, Marshall-Pescini et al. (4, 22) found differences in trained dogs' gazing and persistence patterns, but other authors did not find these differences (18, 24, 31). Results regarding working dogs' abilities should be taken with caution, as dogs from different studies vary in the type and amount of training they have received. For example, dogs in Marshall-Pescini et al. (22) were trained for different purposes (agility, police, search and rescue, and man-trailing), while Brubaker and Udell (24) tested search and rescue dogs, D'Aniello et al. (31) focused on water rescue dogs, and in Topál et al. (18) dogs were trained for basic obedience. A possible explanation is that specific training and working experience confounded the results. We have tried to independently assess (1) the effect of training for recreational purposes as dogs in our trained family group were trained for different hobby activities, mainly obedience and agility, and (2) the effect of specific training, as working dogs were trained as assistance and therapy dogs. Therefore, the type and methods of training could be an important aspect to take into account in future studies. Most probably the broad category “trained vs. untrained” is not precise enough to unravel the effect of training on problem-solving behaviors. Furthermore, working dogs may vary in their independence levels according to the context in which they work. For instance, water rescue dogs did not differ from pet dogs in their interaction with the apparatus during an unsolvable task, but they directed their first gaze significantly more often toward the owner and spent more time gazing at people than untrained pet dogs (31). Water rescue dogs are rewarded for looking at the handler during their training, and during their service they have to remain inactive for a long time in the vicinity of their owners in order not to cause any disturbance, and they take initiatives only upon command. These specific requirements probably affect their performance during problem-solving tasks.

A direct antecedent in the literature is the study of Mongillo et al. (32) who measured dogs' attention toward the owner in untrained family dogs, agility trained dogs, and assisted intervention animals. They assessed the number of gazes and the amount of time dogs spent watching their owner in a baseline condition where the owner walked alone in a room, and in a selective attention test where the owner's movements were mirrored by an experimenter. During the baseline phase, agility dogs shifted their gaze frequently toward the owner and were also the ones who spent the lesser amount of time looking at their owners, while assistance dogs gazed longer. In addition, assistance dogs gazed longer at their owners during the selective attention test. These results support the idea that different training and everyday activities may modify dogs' attentional patterns. Contrary to our results, Mongillo et al. (32) found that dogs participating in animal assisted interventions were the most attentive to their owners. This apparent contradiction could be due to the differences in the task. Unlike Mongillo et al. (32), we presented dogs with a problem-solving situation, in which dogs had to manipulate an apparatus to access a reward. In this latter scenario we observed that working dogs (which include dogs participating in animal assisted interventions) displayed less social strategies than the other group of dogs. Assistant and therapy dogs have to be attentive to their owners' needs but once they understand them or receive a specific command, they should be independent to succeed in their tasks. This interpretation is also supported by the fact that agility dogs in Mongillo et al. (32) shifted their gaze toward the owner more frequently than family dogs which is an important feature in the agility sport, but they do not need to solve novel problems independently during it. In our study trained family dogs (which include agility dogs) differed in the use of social strategies from untrained pet dogs. Thus, training for specific purposes may yield different patterns of social behavior depending on the context, emphasizing the plasticity and adaptability of dogs' behavior.

Importantly, according to the SNK *post-hoc* tests, trained family dogs had lower social strategy scores than untrained family dogs. Possibly, trained dogs were more used to facing novel situations and they could have generalized their training experience to this situation as well. It is possible that during training sessions dogs have to persevere and try different behaviors before getting the reward and that the contextual cues of the testing scenario trigger some of those responses. Indirect evidence supporting this idea comes from studies indicating that dogs are able to generalize and learn to follow novel and complex communicative signals faster when they have previously received a brief training phase with a simpler communicative cue (33, 34).

Nevertheless, training for recreational purposes did not seem to be enough for dogs to reach the effectiveness of working assistance and therapy dogs, as the latter were more successful problem solvers and had lower scores in the use of the social strategies component. This result suggests that dogs' everyday experience is an uttermost important aspect to take into account when assessing their skills in a problem-solving situation. There are at least two possible, non-exclusive, explanations for this difference. First, it is possible that working assistance and therapy dogs were more comfortable in the presence of strangers and in novel situations given that they usually accompany their owners to a variety of places. Second, it is possible that dogs that have successfully accomplished the training as assistance or therapy dogs had pre-existing characteristics that distinguished them from other dogs. For instance, it has been shown that personality traits such as boldness are related with the successful training of working dogs (35). We propose that these two explanations are complementary, because it is possible that those dogs that became working dogs were encouraged during their everyday activities to behave in a more independent manner. Owners were allowed to encourage their dogs during the task, verbally or pointing to the apparatus, but without using commands or touch. Interestingly, Udell (11) reported that dogs, who were encouraged, spent more time in contact and looking at the puzzle box, but they were not significantly more successful in solving the task. Similarly, in Brubaker and Udell (24) encouraged family dogs interacted more with the apparatus but their performance was not significantly better. Conversely, encouragement did improve the performance of dogs trained for search and rescue (24). Given that in the present study we did not systematically manipulate the quantity and quality of the encouragement, we cannot derive unambiguous conclusions regarding this aspect. Udell's (11) results suggest that the use of encouragement and verbal instructions modulates problem-solving behavior, but their particular effects could depend on the context as well as working and training experience (11). In this regard, it is also possible that dogs react differently to verbal commands. Working dogs are trained to respond to a command by performing a specific action. For example, if the owner points to a particular object and asks the dog to do something with it, trained working dogs will manipulate the object instead of looking at the owner, while untrained pet dogs may be uncertain about what to do and will gaze at the owner in search for further clues [similarly to young dogs in Miklósi et al. (5)]. Furthermore, not only the type of commands given by the owner affects dogs' performance, but also the bond between them. Topál et al. (18) compared the performance of dogs categorized according to their relationship with the owners. “Companion dogs” were defined as dogs living indoors as a member of the family and “working dogs” were kept outside the house as a guard or for some other purpose. In a simple manipulative task dogs had to manipulate an apparatus in order to get the reward while the owner could encourage them to retrieve the food. Companion dogs gazed more at the owner, started to manipulate the apparatus later and also retrieved less food than working dogs. The authors also found that obedience training did not affect dogs' performance or gazing patterns to their owners. These results are in line with our findings about the similar gazing patterns between trained and untrained family dogs.

One limitation of the study is that the dogs' characteristics before training were unknown. As it occurs in many studies assessing the effect of training on dogs' cognitive skills, the lack of a baseline measurement before training makes it impossible to guarantee that dogs were not selected for such work based on their pre-existing characteristics such as an increased persistence. Another limitation of these kind of studies is that training methods may differ between specific trainers and yield different results on dogs' problem-solving strategy. Thus, in future research, specific types and methods of previous training should also be taken into account when assessing dogs' problem-solving skills.

Summing up, we have shown that working assistance and therapy dogs were more independent problem solvers compared to both trained and untrained family dogs, who privileged a more social strategy. Thus, although assistance and therapy dogs need to show highly developed social understanding in their interactions with the owner, their special training and work may have increased their persistence and independent problem-solving skills. However, obtaining training certificates (mainly obedience and agility in this study) also increased the independent problem-solving tendency in our task, suggesting that trained family dogs generalize their training experience of facing novel situations and perseverance for obtaining rewards.

## Data Availability Statement

All datasets generated for this study are included in the article/Supplementary Material.

## Ethics Statement

The animal study was reviewed and approved by Pest County Government Office, PE/EA/2019-5/2017. Written informed consent was obtained from the owners for the participation of their animals in this study.

## Author Contributions

EK, ÁM, and MG designed the experiments and collected the data. EK analyzed the data. FC, CC, and EK wrote the first draft. All authors finalized the manuscript. ÁM and EK provided funding.

### Conflict of Interest

The authors declare that the research was conducted in the absence of any commercial or financial relationships that could be construed as a potential conflict of interest.
